# Validation of a portable monitor compared with polysomnography for screening of obstructive sleep apnea in polio survivors

**DOI:** 10.3389/fneur.2023.1137535

**Published:** 2023-05-09

**Authors:** Qidi Ding, Jianghua Liu, Jinxian Wu, Juan Du, Xiao Li, Meng Wang, Yunliang Sun, Yan Yu, Jingyu Wang, Ting Sun, Chi Zhang, Changjun Lv, Kingman P. Strohl, Fang Han, Xiaosong Dong

**Affiliations:** ^1^Department of Respiratory and Sleep Medicine, Peking University People's Hospital, Beijing, China; ^2^Department of Respiratory and Critical Care Medicine, Binzhou Medical University Hospital, Binzhou, Shandong, China; ^3^Department of Pediatric, Dongyang People's Hospital, Jinhua, Zhejiang, China; ^4^Department of Sleep Medicine, Dongyang Seventh People's Hospital, Jinhua, Zhejiang, China; ^5^Department of the First School of Clinical Medicine, Binzhou Medical University, Binzhou, Shandong, China; ^6^Division of Pulmonary, Critical Care and Sleep Medicine, Department of Medicine, Case Western Reserve University, and Cleveland Louis Stokes VA Medical Center, Cleveland, OH, United States

**Keywords:** sleep-disordered breathing, obstructive sleep apnea, postpolio, neuromuscular disorder, portable monitor

## Abstract

**Subjective:**

Sleep-disordered breathing (SDB) is highly prevalent in polio survivors. Obstructive sleep apnea (OSA) is the most frequent type. Full polysomnography (PSG) is recommended for OSA diagnosis in patients with comorbidities by current practice guidelines, but it is not always accessible. The purpose of this study was to evaluate whether type 3 portable monitor (PM) or type 4 PM might be a viable alternative to PSG for the diagnosis of OSA in postpolio subjects.

**Methods:**

A total of 48 community-living polio survivors (39 men and 9 women) with an average age of 54.4 ± 5.3 years referred for the evaluation of OSA and who volunteered to participate were recruited. First, they completed the Epworth Sleepiness Scale (ESS) questionnaire and underwent pulmonary function testing and blood gas tests the day before PSG night. Then, they underwent an overnight in-laboratory PSG with a type 3 PM and type 4 PM recording simultaneously.

**Results:**

The AHI from PSG, respiratory event index (REI) from type 3 PM, and ODI_3_ from type 4 PM was 30.27 ± 22.51/h vs. 25.18 ± 19.11/h vs. 18.28 ± 15.13/h, respectively (*P* < 0.001). For AHI ≥ 5/h, the sensitivity and specificity of REI were 95.45 and 50%, respectively. For AHI ≥ 15/h, the sensitivity and specificity of REI were 87.88% and 93.33%, respectively. The Bland–Altman analysis of REI on PM vs. AHI on PSG showed a mean difference of −5.09 (95% confidence interval [CI]: −7.10, −3.08; *P* < 0.001) with limits of agreement ranging from −18.67 to 8.49 events/h. ROC curve analysis for patients with REI ≥ 15/h showed an area under the curve (AUC) of 0.97. For AHI ≥ 5/h, the sensitivity and specificity of ODI_3_ from type 4 PM were 86.36 and 75%, respectively. For patients with AHI ≥ 15/h, the sensitivity was 66.67%, and the specificity was 100%.

**Conclusion:**

Type 3 PM and Type 4 PM could be alternative ways to screen OSA for polio survivors, especially for moderate to severe OSA.

## 1. Introduction

Reduced diaphragmatic strength and respiratory muscle weakness contribute to the development of sleep-disordered breathing (SDB) in patients with neuromuscular disorders (NMD) (1). Obstructive sleep apnea (OSA) is very frequent in patients with NMD (2). Predisposing factors include upper airway (oropharyngeal) muscle weakness, macroglossia, male sex, and obesity (3). Polio survivors, as a special type of NMD, also have a high incidence of SDB (4–6). It is estimated that there are 15–20 million polio survivors worldwide and approximately 2.8 million polio survivors in China in 2006 (7). The frequency of SDB in polio survivors ranged from 7.3 to 65% (8, 9). The most frequent SDB among polio survivors is OSA (10). Since effective polio vaccination was used since the 1950s in China, the majority of Chinese polio survivors are now middle-aged to elderly, which is a high risk of OSA. These patients with OSA represent a significant proportion of the population and raise concerns about the public health burden.

Early detection of OSA and timely intervention may prevent morbidity and improve the quality of life in patients with NMD. Full polysomnography (PSG) is currently considered the gold standard for SDB diagnosis. However, PSG has some disadvantages, such as high cost, long waiting time, and lack of barrier-free equipment, especially in developing countries. Current clinical practice guidelines have recommended the use of portable monitors (PMs) for the diagnosis of OSA in adults (11). An increasing number of studies have already proven the diagnostic accuracy of PM for OSA with comorbidities, including some kinds of NMDs (12–14). Type 3 PM studies use devices that measure limited cardiopulmonary parameters, two respiratory variables (e.g., an effort to breathe and airflow), oxygen saturation, and a cardiac variable (e.g., heart rate or electrocardiogram). Type 4 PM studies utilize devices that measure only 1 or 2 parameters, typically oxygen saturation and heart rate, or in some cases, just airflow (11). It is reported that the sensitivity and specificity of type 3 PM for detecting AHI ≥5 events/hour on PSG ranged from 33 to 100% and 9 to 100%, respectively. The sensitivity and specificity of pulse oximetry ODI ranged from 48 to 97% and 63 to 100%, respectively, for PSG AHI/RDI cutoff values of 5–15 events/h. In general, both sensitivity and specificity improved with an increased number of parameters measured although they varied across different devices (15). However, there has been no validation study of PMs for OSA in postpolio patients till now. The purpose of the current study was to evaluate whether a type 3 PM (Nox-T3, Nox Medical Inc., Reykjavik, Iceland) or a type 4 PM (Pulsox-300i pulse oximetry, KONICA MINOLTA, Osaka, Japan) might be a viable alternative to PSG for the diagnosis of OSA in postpolio subjects.

## 2. Methods

### 2.1. Study design and data collection

#### 2.1.1. Participants

From June 2019 to November 2019, we performed this study in Dongyang City, Zhejiang Province, China. Potential postpolio survivors, as defined by polio exposure of >25 years earlier, were identified in Dongyang City with the help of the local office of the Organization for Disabled People, a national agency to assist those with chronic disabling conditions. Participants were excluded if they had sleep studies with unusable physiologic parameters or <4 h of sleep time, medical treatment for SDB, or physician-diagnosed stroke or cardiovascular disease. The calculation of sample size was provided in the Supplementary material. The study was approved by the institutional review board of Binzhou Medical University. Written informed consent was obtained from all participants.

#### 2.1.2. Measurements

All participants completed the Epworth Sleepiness Scale (ESS) questionnaire and underwent pulmonary function testing and blood gas tests the day before PSG night. They underwent an overnight in-laboratory PSG (Alice 6, Philips Respironics, Inc., United States) with simultaneous Nox-T3 PM recording and Pulsox-300i pulse oximetry PM recording applied by the PSG technician. The nasal pressure recording was split using a Y connector with one limb going to the PSG system and the other to the Nox-T3 PM system.

Standard full PSG channels were recorded according to the American Academy of Sleep Medicine (AASM) recommendations (16). The following signals were recorded: electroencephalogram (F3M2, F4M1, C3M2, C4M1, O1M2, and O2M1), bilateral electrooculogram, chin muscle electromyogram, oronasal thermistor, nasal pressure, rib cage and abdominal movement, electrocardiogram, snoring, and body saturation by pulse oximetry. Scoring of PSG recordings was performed by a single experienced, certified PSG technologist using standard AASM criteria. The apnea-hypopnea index (AHI) was defined as the number of apneas and hypopneas (based on 3% desaturation) per hour of sleep. The oxygen desaturation index (ODI_3_) was defined as the number of oxygen desaturation >3% per hour of sleep.

Nox-T3 is a type 3 PM with nasal pressure airflow, thoracic and abdominal movement, snoring, body position, pulse rate, and oxygen saturation signals by pulse oximetry. Nox-T3 PM was installed for simultaneous recording during PSG by the PSG technician in the laboratory. The PM recordings were scored by an experienced technologist according to the AASM criteria, who was blinded to the PSG result. The PM start and stop times were determined according to the changes in the audio and body motion signals in the subject's recorded data, and the total analysis time (TAT) was obtained. Then, the respiratory event index (REI) was calculated, which was defined as the number of apneas and hypopneas (based on 3% oxygen desaturation) per hour of TAT. If no adequate pulse oximetry, effort, and airflow signals were available or the recording time was < 4 h, the recording was determined to fail. If some signals were suboptimal or missing but the study was interpretable, then the recording was classified as partially successful.

Pulsox-300i pulse oximetry is a type 4 PM with pulse rate and oxygen saturation signals. Pulsox-300i PM was installed for simultaneous recording during PSG by the PSG technician in the laboratory. The oximetry data were calculated using the self-calculating program.

### 2.2. Statistical analysis

Continuous variables are summarized using the mean and standard deviation (SD), and categorical variables are summarized using count and percentage. Student's *t*-test was used to compare normally distributed continuous data, while the Mann–Whitney U-test was performed when continuous data were not normally distributed. Categorical variables were compared using the chi-square test. Comparisons of respiratory parameters among the three monitoring methods were compared using a one-way ANOVA analysis.

To compare the diagnostic ability of PM vs. PSG for OSA in polio survivors, we calculated the sensitivity, specificity, positive predictive value, and negative predictive value at AHI thresholds of ≥5, ≥15, and ≥30 events/h for each PM method, using the results of the in-laboratory PSG as the gold standard.

To assess the level of agreement between the monitoring methods, we used paired tests and methods described by Bland and Altman (17). Specifically, for a given metric (e.g., AHI), we first calculated the participant-specific difference for each pair of methods and tested whether this was significantly different from zero using paired *t*-tests. Next, for each pair of monitoring methods, we examined the relationship between the participant-specific difference and the participant-specific average value using the two techniques. This relationship was evaluated graphically and statistically for bias, including examining the average participant-specific difference and associated limits of agreement (equal to the mean difference ± 2 SD) and testing for a significant correlation between the participant-specific difference and mean (e.g., whether differences between techniques are larger/smaller for higher/lower average values). Correlation plots were constructed to visually compare REI and ODI_3_ from PM vs. AHI from PSG as PM had relatively high-diagnostic accuracy for moderate to severe OSA in the general population (11). Receiver operating characteristic (ROC) curves were only constructed for REI and ODI_3_ from PM at AHI thresholds of 15/h to determine the diagnostic threshold of moderate and severe OSA. The best predictive threshold value from the PM was calculated that optimized sensitivity and specificity for the detection of PSG-based threshold severity.

All analyses were performed using SPSS version 20.0 and MedCalc. A value of *P* < 0.05 was used to determine statistical significance.

## 3. Results

### 3.1. Sample characteristics

**Figure 1:**
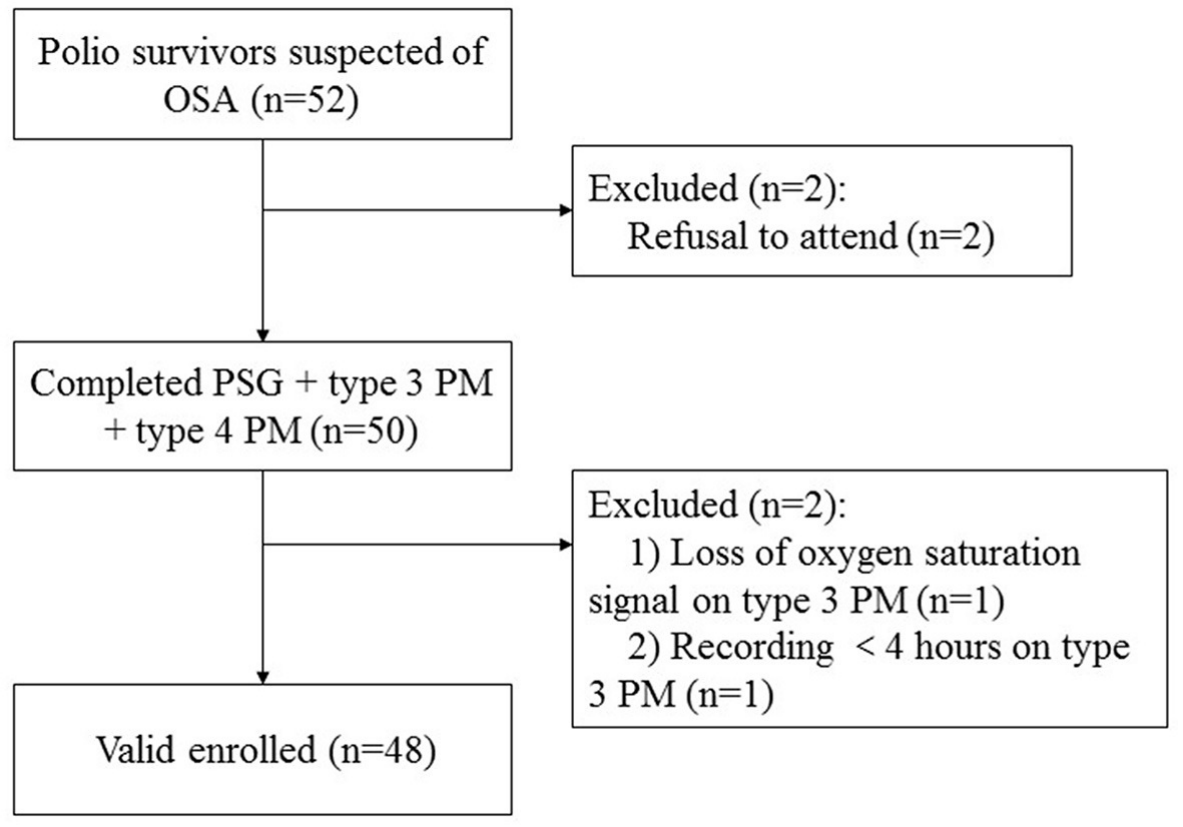
Flow chart of participants. OSA, obstructive sleep apnea; PM, portable monitor; PSG, polysomnography.

Of the 52 polio survivors referred for this study, two declined to participate, and two subjects were considered unsuccessful due to the type 3 PM being unable to be interpreted, resulting in 48 enrolled adult subjects (Figure 1). The enrolled participants were middle-aged (54.38 ± 5.16 years) with a polio infection history of 51.37 ± 4.67 years, were normal weight (mean body mass index (BMI) 24.97 ± 3.22 kg/m^2^, where seven of them have BMI >28 kg/m^2^), and were predominantly male (79.2%). In total, 25% of the subjects reported being sleepy with ESS scores >10 points and 61.36% of them reported snoring. Ten (20.83%) subjects had a forced vital capacity (FVC) < 80% of the predicted value. The mean FVC was 94.02 ± 16.50%, the mean FEV1/FVC was 99.82 ± 7.52%, and the mean RV was 95.34 ± 19.31%. The blood gas test showed that the mean PO_2_ was 82.21 ± 20.32 mmHg and the mean PCO_2_ was 38.54 ± 4.08 mmHg.

### 3.2. Comparison of respiratory parameters

**Figure 2:**
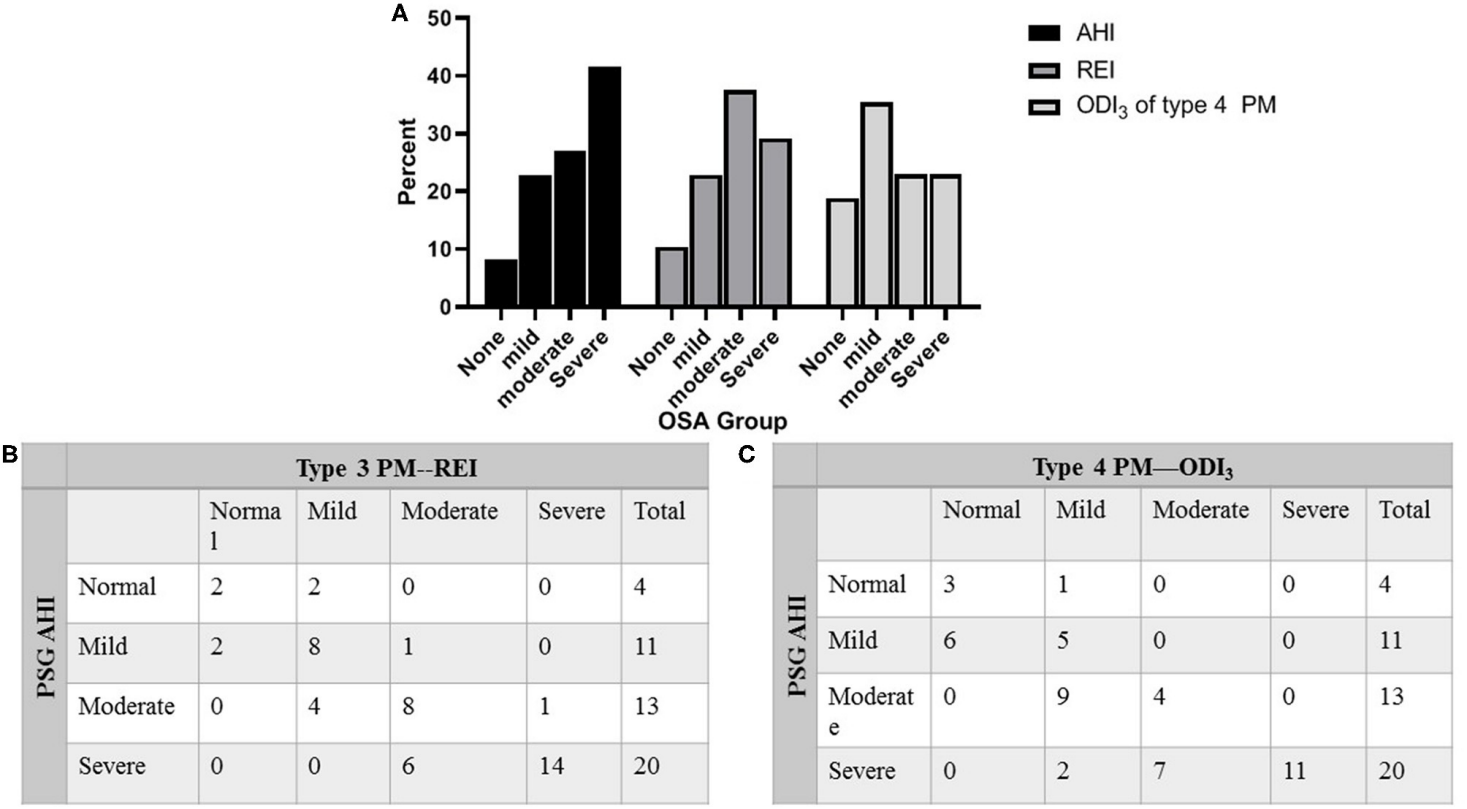
Percentage of patients categorized into clinical OSA groupings. **(A)** Percentage of patients categorized into clinical OSA groupings of none (AHI < 5 events/h), mild (AHI 5 to < 15 events/h), moderate (AHI 15 to < 30 events/h), and severe (AHI ≥ 30 events/h) based on AHI of PSG, REI of type 3 PM, and ODI_3_ of type 4 PM. **(B)** Distribution of total subjects evaluated by PSG against type 3 PM. **(C)** Distribution of total subjects evaluated by PSG against type 4 PM. AHI, apnea–hypopnea index; REI, respiratory event index; ODI_3_, oxygen desaturation >3% index; OSA, obstructive sleep apnea; PM, portable monitor; PSG, polysomnography.

Table 1 compares the results of PSG, type 3 PM, and type 4 PM. There was no difference between PSG and type 3 PM in the number of total respiratory events per night (181.79 ± 131.98 vs. 187.33 ± 145.49, *P* = 0.41). The total sleep time (TST) was shorter on PSG than TAT on type 3 PM and total recording time (TRT) on type 4 PM, *P* < 0.001). The AHI on PSG was slightly higher than REI on type 3 PM (30.27 ± 22.51 vs. 25.18 ± 19.11 events/h, P < 0.001). As the longest total recording time and limited recording signal, the ODI_3_ of type 4 PM was significantly lower than REI and AHI (*P* < 0.001). Statistically significant differences in oxygen desaturation severity measures were observed among the three methods as reported in COPD patients (14). Figure 2 illustrates that six subjects (30%) of the severe group categorized into the moderate group in type 3 PM, and seven subjects (35%) of the severe group categorized into the moderate group and two subjects (10%) categorized into the mild group in type 4 PM.

**Table 1:** PSG, type 3 PM, and type 4 PM data for the study participants.

	**PSG**	**Type 3 PM**	**Type 4 PM**	***P***
Total sleep time/TAT (min)	366.07 ± 55.87	449.04 ± 53.32	477.13 ± 29.25	< 0.001^*^
**Respiratory events**				
Total respiratory events	181.79 ± 131.98	187.33 ± 145.49	NA	0.41#
AHI/REI (events/h)	30.27 ± 22.51	25.18 ± 19.11	NA	< 0.001#
OSA index (events/h)	9.95 ± 15.58	10.25 ± 14.97	NA	0.61#
MSA index (events/h)	3.1 ± 5.83	1.61 ± 3.22	NA	0.009#
CSA index (events/h)	0.78 ± 1.07	1.08 ± 1.14	NA	0.023#
Hypopnea index (events/h)	16.44 ± 12.60	12.24 ± 9.43	NA	< 0.001#
ODI_3_ (events/h)	18.69 ± 18.55	24.36 ± 19.44	18.28 ± 15.13	< 0.001^*^
Mean SpO_2_ (%)	95.98 ± 1.45	94.05 ± 1.69	94.90 ± 1.38	< 0.001^*^
Lowest SpO_2_ (%)	83.02 ± 8.22	81.02 ± 7.95	78.07 ± 10.49	< 0.001^*^

PSG, polysomnography; PM, portable monitor; TAT, total analysis time; AHI, apnea hypopnea index; REI, respiratory event index; OSA, obstructive sleep apnea; MSA, mixed sleep apnea; CSA, central sleep apnea; ODI_3_, oxygen desaturation > 3% index.

* Comparisons of respiratory parameters among PSG, type 3 PM, and type 4 PM. #Comparisons of respiratory parameters between PSG and type 3 PM.

### 3.3. Detection of OSA by type 3 PM and type 4 PM in postpolio patients

Cross tabulation of the REI and ODI_3_ by AHI is shown in Table 2. The REI performance for detecting OSA (AHI≥5/h) showed a sensitivity of 95.45% and a specificity of 50%. For detecting subjects with AHI ≥ 15/h, the sensitivity was 87.88%, and the specificity was 93.33%. For patients with AHI ≥ 30/h, the sensitivity was 70%, and the specificity was 96.43% (Table 3). The performance of ODI_3_ on type 4 PM compared to AHI on PSG is shown in Table 3. For subjects with AHI ≥5/h, the sensitivity was 86.36% and the specificity was 75%. For patients with AHI ≥ 15/h, the sensitivity was 66.67% and the specificity was 100%.

**Table 2:** Cross-tabulation of the REI and ODI_3_ by AHI.

		**Total**	**Positive (%)**	**Negtive (%)**
Type 3 PM	AHI≥5/h	44	REI≥5/h	REI < 5/h
			42 (95.45)	2 (4.55)
	AHI≥15/h	33	REI≥15/h	REI < 15/h
			29 (87.88)	4 (12.12)
	AHI≥30/h	20	REI≥30/h	REI < 30/h
			14 (70)	6 (30)
Type 4 PM	AHI≥5/h	44	ODI_3_≥5/h	ODI_3_ < 5/h
			38 (86.36)	6 (13.64)
	AHI≥15/h	33	ODI_3_≥15/h	ODI_3_ < 15/h
			22 (66.67)	11 (33.33)
	AHI≥30/h	20	ODI_3_≥30/h	ODI_3_ < 30/h
			11 (55)	9 (45)

AHI, apnea hypopnea index; REI, respiratory event index; ODI_3_, oxygen desaturation>3% index; PM, portable monitor.

**Table 3:** Performance of REI and ODI_3_ of type 4 PM vs. AHI for different cutoffs.

	**Percentage**	**Sensitivity (%) (95%CI)**	**Specificity (%) (95%CI)**	**PPV (%) (95%CI)**	**NPV (%) (95%CI)**
		**Type 3 PM**			
AHI≥5/h	91.67%	95.45 (83.3, 99.21)	50 (9.19, 90.81)	95.45 (83.3, 99.21)	50 (9.19, 90.81)
AHI≥15/h	68.75%	87.88 (70.86, 96.04)	93.33 (66.03, 99.65)	96.67 (80.95, 99.83)	77.78 (51.92, 92.63)
AHI≥30/h	41.67%	70 (45.67, 87.16)	96.43 (79.76, 99.81)	93.33 (66.03, 99.65)	81.82 (63.92, 92.38)
**Type 4 PM**					
AHI≥5/h	91.67%	86.36 (71.95,94.33)	75 (21.94,98.68)	97.44 (92.48,100)	33.33 (2.5,64.13)
AHI≥15/h	68.75%	66.67 (48.10,81.45)	100 (74.65,100)	100 (100,100)	57.69 (38.70,76.68)
AHI≥30/h	41.67%	55 (32.05,76.17)	100 (84.98,100)	100 (100,100)	75.68 (61.85,89.5)

PM, portable monitor; REI, respiratory event index; ODI_3_, oxygen desaturation>3% index; AHI, apnea-hypopnea index; PPV, positive predictive value; NPV, negative predictive value.

**Figure 3:**
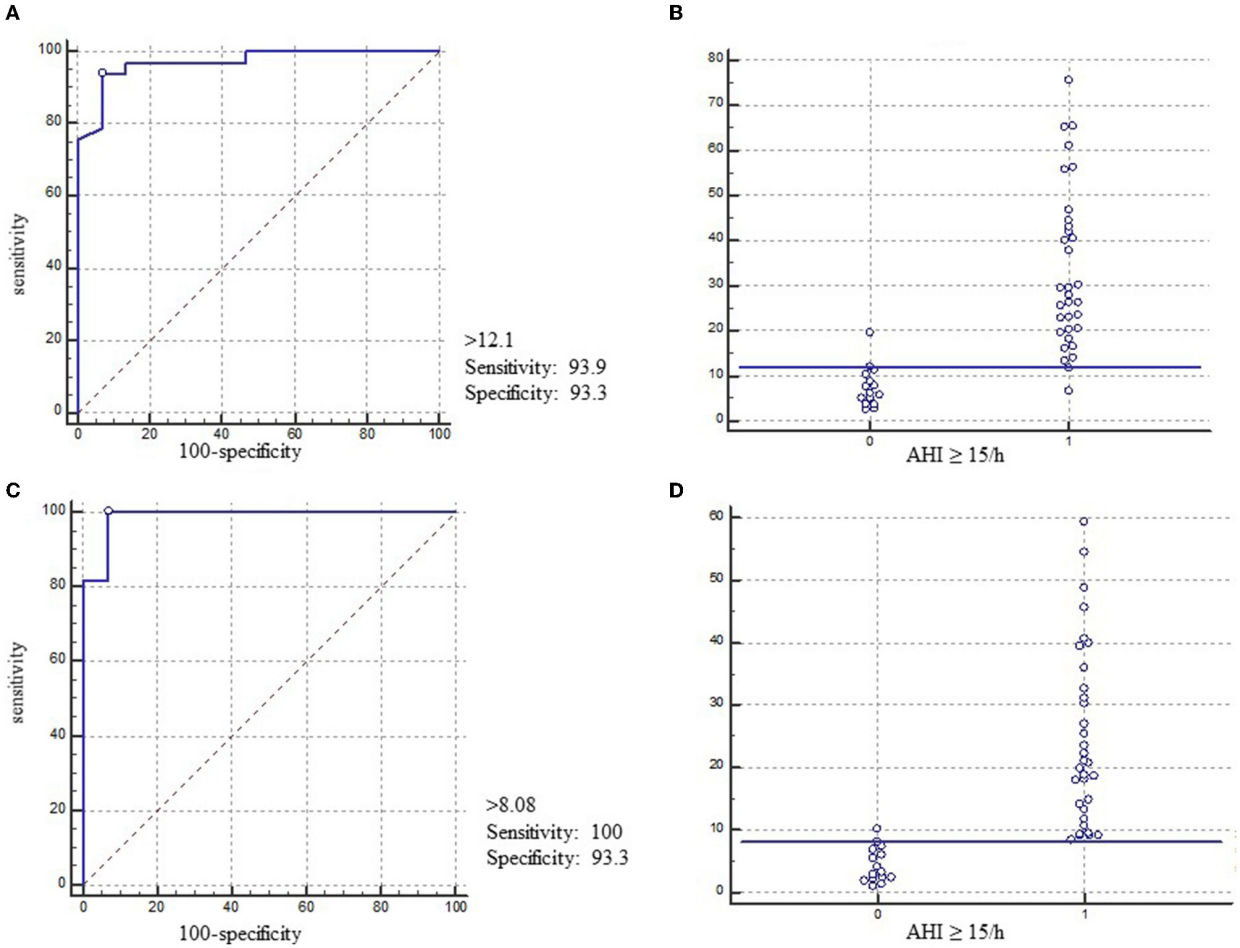
ROC curve and performance graphs for type 3 PM and type 4 PM compared to PSG. ROC curve **(A)** and performance graphs **(B)** for AHI ≥15/h of REI from type 3 PM variables and ROC curve **(C)** and performance graphs **(D)** for AHI ≥15/h of ODI_3_ from type 4 PM variables. AHI, apnea-hypopnea index; AUC, area under the curve; REI, respiratory event index; ODI_3_, oxygen desaturation >3% index; PM, portable monitor.

The ROC curve analysis for patients with REI ≥ 15/h showed an area under the curve (AUC) of 0.97, 95% CI: 0.88–1.00 (Figure 3A). For AHI ≥15/h, the optimal cutoff of REI was 12.1/h (Youden index 0.87, sensitivity 93.94%, and specificity 93.33%). The ROC curve analysis for patients with ODI_3_ from type 4 PM ≥15/h showed an AUC of 0.98, 95% CI: 0.90–1.00 (Figure 3C). For AHI ≥15/h, the optimal cutoff of ODI_3_ was 8.1/h (Youden index 0.93, sensitivity 100%, and specificity, 93.33%).

### 3.4. Agreement of AHI between monitoring methods

**Figure 4:**
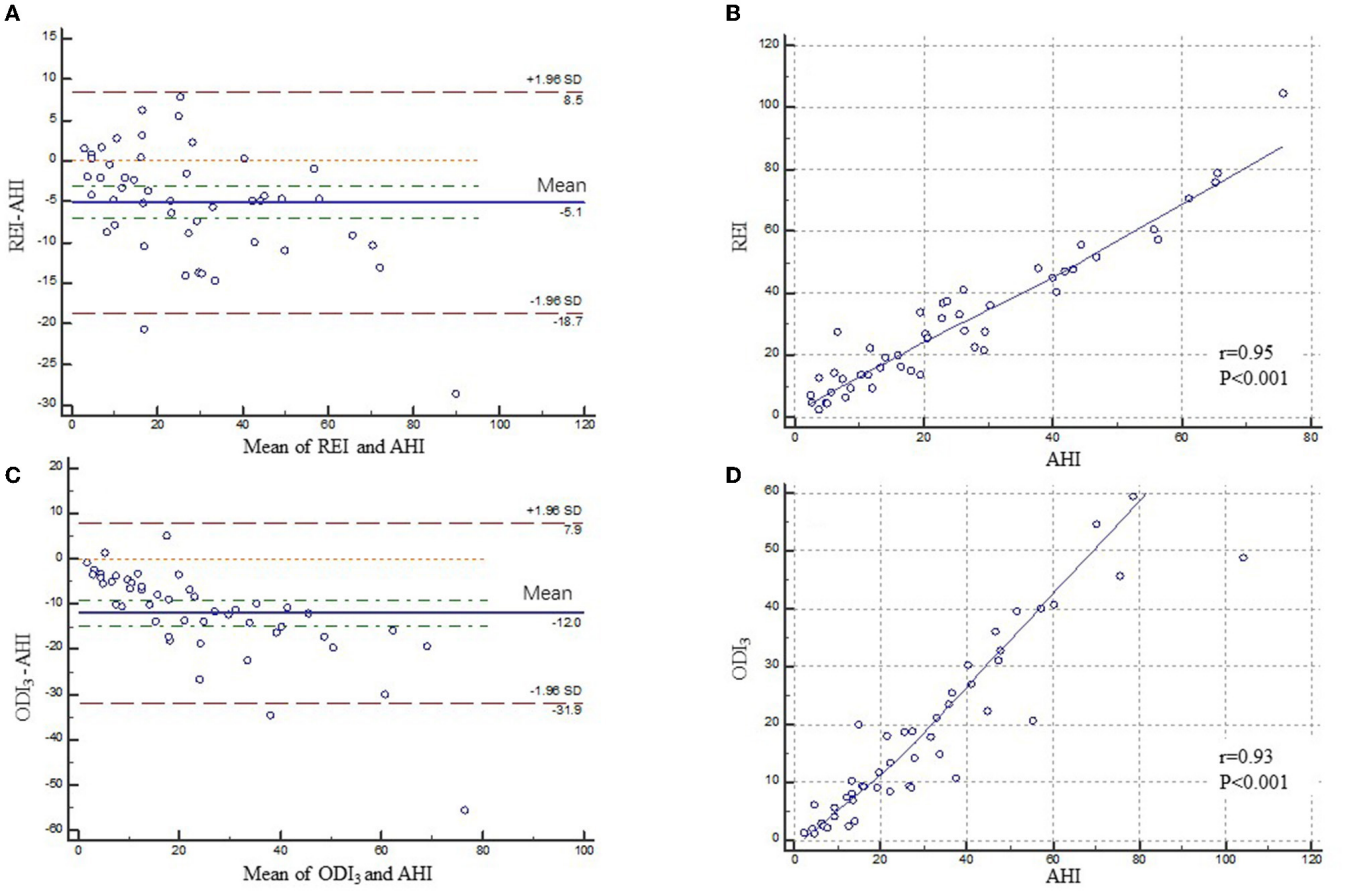
Bland–Altman and correlation plots for type 3 PM and type 4 PM compared to PSG. Bland–Altman **(A)** and correlation plots **(B)** comparing AHI from PSG and REI from type 3 PM. Bland–Altman **(C)** and correlation plots **(D)** comparing AHI from PSG and ODI_3_ from type 4 PM. PM, portable monitor; PSG, polysomnography; AHI, apnea-hypopnea index; REI, respiratory event index; ODI_3_, oxygen desaturation>3% index.

Bland–Altman and identity plots comparing REI of type 3 PM and ODI_3_ of type 4 PM with AHI of PSG are shown in Figure 4. The Bland–Altman analysis of REI versus AHI showed a mean difference of −5.09 (95% confidence interval [CI]: −7.10, −3.08; *P* < 0.001) with limits of agreement ranging from −18.67 to 8.49 events/h. The correlation coefficient for type 3 PM was 0.96 on the AHI vs. REI identity plot (*P* < 0.001). The Bland–Altman analysis of ODI_3_ on type 4 PM vs. AHI on PSG showed a mean difference of −11.99 (95% confidence interval [CI]: −14.93, −9.04; *P* < 0.001) with limits of agreement ranging from −31.85 to 7.88 events/h. The correlation coefficient was 0.93 on the AHI vs. ODI_3_ on the type 4 PM identity plot (P < 0.001). The 95% limits of agreement were wider for ODI_3_ on type 4 PM than for REI.

## 4. Discussion

This is the first study on OSA diagnosis validation of PMs in a postpolio cohort. We found that both type 3 PM and type 4 PM are feasible for detecting OSA in polio survivors. REI from type 3 PM had relatively higher sensitivity in diagnosing OSA than that of ODI_3_ from type 4 PM. REI also had better correlation and agreement with PSG to identify OSA. Cutoffs for AHI ≥15 could be achieved so that sensitivity and specificity of type 3 PM and type 4 PM are good to identify moderate to severe OSA in this special population.

We found that either REI or ODI_3_ was lower than AHI. However, there is no significant difference in total respiratory events between PSG and type 3 PM. Type 3 PM or type 4 PM does not record EEG signals to measure TST accurately, so the TAT/TRT used to calculate REI or ODI_3_ in PM is significantly longer than TST. Without EEG, type 3 PM lacked the ability to detect arousals, so the hypopneas based on an associated arousal would be missed. Moreover, we speculate that these differences may also have been due to the difference in flow detection sensitivity between PSG and type 3 PM. PSGs use both thermal and pressure excursion to determine the grade of an obstructive event, while type 3 PM uses only pressure. Nonetheless, this difference did not have a major influence on clinical decision-making, as type 3 PM only missed a few patients with moderate to severe OSA. This is also reported by studies on type 3 PM in OSA with and without comorbidities (13, 14, 18). In studies on type 4 PM, though different oximetry devices were performed variously, the newly developed technology with accelerometer and PPG analysis may decrease the disparity between TRT and TST (19, 20). However, they were only validated in OSA patients without comorbidities.

In our study, the sensitivity of REI was not as high as that of Chinese OSA patients without comorbidities, which is similar to the results of OSA combined with COPD and chronic heart failure (13, 14). We consider that it was mainly associated with the SDB characteristics of NMD. Patients with NMD had more hypopnea events, while type 3 PM had no EEG signal to identify arousals caused by hypopnea (21). This was also reported by Westenberg et al. in which the sensitivity for AHI ≥15/h is 64% in patients with various kinds of NMD (12). Both studies found that hypopnea is the major event during sleep, which would be underestimated by type 3 PM. The sensitivity of ODI_3_ on type 4 PM is relatively low, but the specificity is high. Hence, for type 4 PM, the specificity and PPV are of clinical significance to start intervention in patients with moderate to severe OSA. The sensitivity of REI on type 3 PM was higher than that of ODI_3_ on type 4 PM, and the mean difference value of the consistency test was also lower than that of ODI_3_. Compared with ODI_3_, REI obtained more information from nasal pressure and thoracic/abdominal movement other than only SpO_2_, so that it could identify more apnea events without oxygen desaturations. When the patient's basal SpO_2_ is low, especially in patients with NMD or severe OSA, REI can identify respiratory events more sensitively. On the other hand, ODI_3_ on type 4 PM had relatively high specificity. It indicated that ODI_3_ also had a screening value for OSA in postpolio patients. For potentially hypoxic patients due to NMD, Rodrigues Filho et al. used different degrees of baseline SpO_2_ drop (3, 4, and 5%) and created a time criterion for the duration of desaturation, and then they found ODI_3/2_ (ODI_3_ with the duration of 2 s) has the best sensitivity and specificity (22). Unfortunately, the software of Pulsox-300i could not provide such variables, so we could not repeat their findings.

The SpO_2_ monitored by type 3 or type 4 PM was lower than the corresponding PSG monitoring value. Previous verification tests have also found a similar problem (13, 14). The main reason is the difference between different sensors. However, in this study, even if there was a slight difference in SpO_2_ monitoring, the consistency of ODI_3_ on PM to PSG was good.

The AUC of REI ≥ 15/h was 97%, which is within the reported AUC ranges published before in the general population (23). The AUC of ODI_3_ ≥ 15/h was 98% which is higher than that of REI. ODI_3_ was proven to be a diagnostic tool for OSA, even in patients with NMD and potentially hypoxic diseases (22). However, due to low sensitivity, underestimation of AHI, and inability to distinguish different SDB types, REI from type 3 PM and ODI_3_ from type 4 PM alone are not adequate measures to diagnose SDB in postpolio subjects. For the identification of a patient of AHI ≥ 15/h, the optimal cutoff of REI was 12.1/h, while that of ODI_3_ was 8.1/h, which would balance the sensitivity and specificity for moderate to severe OSA. Our results are consistent with the findings of Westenberg et al. and Rodrigues Filho et al. (12, 22).

The Bland–Altman analysis of REI vs. AHI showed a mean difference of −5.09 with limits of agreement ranging from −18.67 to 8.49 events/h in polio survivors, while it is reported that in the Bland–Altman plot of AHI on NOX-T3 PM vs. PSG, the mean difference was only −1.4 with narrower limits of agreement of −12.0 to 9.2 events/h in Chinese general adults (18). We can see that both mean differences and limits of agreement in polio patients were greater than those of the general population. Of the 25 studies that evaluated the Bland–Altman concordance analyses, most (n = 19) reported underestimation of AHI using type 3 PM (mean of differences: 15.2 to 24 events/h) in the general population (15). Moreover, wider limits of agreement (−31.85 to 7.88 events/h) and a larger estimated difference (-11.99 events/h) were observed between type 4 PM and PSG in polio patients.

There are few studies that directly compare PM with PSG for postpolio patients. The current guidelines do not recommend the use of type 3 PM or type 4 PM in the diagnosis of OSA for patients with NMD primarily due to the diversity of NMD and lack of high-quality evidence (11). A review on SDB in DMD found three studies reporting PM in DMD and other NMD. The utility of PM in DMD is unclear with only one study to date comparing PSG to PM data (24). Recently, Westenberg JN et al. tested the role of type 3 PM in detecting OSA in patients with NMD, which has been shown to be reliable but somewhat with lower sensitivity (50%) and specificity (88%) (12). Due to inconvenient movement and limited financial capacity, type 3 PM is a more accessible way to diagnose OSA for polio survivors. Type 4 PM has the advantage of easy set-up and a high success rate, also leading it to be an alternative way to screen OSA for polio survivors (19, 22). The results of our current study also provide supportive evidence for the use of type 3 and type 4 PMs.

The participants in this study have good lung function and normal blood gas analysis while awake. As the disease progresses, the weaker the respiratory muscle is, the greater the proportion of alveolar hypoventilation that accounts for patients with NMD (25). Unfortunately, due to the lack of PCO_2_ monitoring, the PM could not demonstrate the SDB in the form of alveolar hypoventilation for postpolio patients. Therefore, for NMD patients who were suspected of sleep hypoventilation, PSG with PCO_2_ monitoring is preferred (21).

There are several limitations to this study. First, due to the inconvenience of postpolio survivors, the number of subjects who completed the test was limited. Second, the subjects were enrolled from community residents without major respiratory muscle weakness. Hence, our results may not be applicable to those with more severe respiratory impairment. Third, polio survivors enrolled in the community generally cannot complete home sleep apnea testing alone because of their education level and physical disability. However, it has been proven that PM as home sleep apnea testing is feasible and reliable for patients with other comorbidities (13, 14). It is convinced that with the help of technicians or family members, PM monitoring that could be taken out of the laboratory will still provide them much convenience. Due to the SPO_2_ differences between devices, it was not possible to determine which pulse oximeter provided the most accurate determination of overnight arterial blood oxygen saturation. It is imperative that the measurement of SpO_2_ be standardized across manufacturers.

In conclusion, this study validates the use of type 3 PM and type 4 PM to diagnose OSA in the postpolio cohort. We found that type 3 PM and Type 4 PM are alternative ways to screen OSA for polio survivors, especially for moderate to severe OSA.

## Data availability statement

The raw data supporting the conclusions of this article will be made available by the authors, without undue reservation.

## Ethics statement

The studies involving human participants were reviewed and approved by the institutional review board of Binzhou Medical University. Written informed consent to participate in this study was provided by the participants' legal guardian/next of kin.

## Author contributions

QD collected data and wrote and reviewed the manuscript. JL, JWu, JD, XL, MW, YS, YY, JWa, and TS collected data. FH and XD designed the research plan and analysis strategy. CZ analyzed the data. FH, CL, and KS revised the manuscript. All authors contributed to the manuscript and approved the submitted version.
