# Supplementary material for: Validation of a portable monitor compared with polysomnography for screening of obstructive sleep apnea in polio survivors
Source: Front Neurol. 2023 May 9;14:1137535. doi: 10.3389/fneur.2023.1137535 (PMC10203206; doi:10.3389/fneur.2023.1137535)
Supplement: Supplementary file 1 [file Data_Sheet_1.PDF]

The sample size calculation in this study used the formula for the diagnostic test.

1) Sample size required for sensitivity evaluation n1

$$n_1 = \frac{Z_{\alpha/2}^2 \times se \times (1 - se)}{d_{Se}^2}$$

2) Sample size required for evaluating specificity n2

$$n_2 = \frac{Z_{\alpha/2}^2 \times sp \times (1 - sp)}{d_{Sp}^2}$$

In the formula α is the inspection level, taken as 0.05 in the calculation;
Z α/ 2 is the cumulative probability of normal distribution, which is 1.96 in the calculation;
Se and Sp respectively represent the estimated values of sensitivity and specificity, d_{Se} is the allowable error of sensitivity, and d_{Sp} is the allowable error of sensitivity (Usually half the width of sensitivity and specificity confidence interval, respectively).
After reviewing the relevant research of classic PM equipment, the same type of PM and the PM used in NMD patients following values of relevant parameters are obtained in this study (Khor YH et al. Sleep Med Rev, 2022) (Xue LY et al. JCSM, 2017) (Rodrigues Filho, JC et al. Sleep Med, 2023) (Westenberg, JN et al. JCSM, 2021). For n1, α taken as 0.05(Z α/ 2=1.96), Se was 0.9, 95%CI was 20% (dSe=0.1). For n2, α taken 0.05(Z α/ 2=1.96), Sp was 0.8, 95%CI was 20% (dSp=0.1). After calculation by the above formula, 35 cases was needed in group n1 and 62 cases was needed in group n2. However, post-polio patients were scattered all over China and had difficulty to move around, only 48 patients were actually enrolled.

Reference:

1. Khor YH, Khung SW, Ruehland WR, et al. Portable evaluation of obstructive sleep apnea in adults: A systematic review. Sleep Med Rev. 2023;68:101743. doi:10.1016/j.smrv.2022.101743

2. Xu LY, Han F, Keenan BT, et al. Validation of the Nox-T3 Portable Monitor for Diagnosis of Obstructive Sleep Apnea in Chinese Adults. Journal of Clinical Sleep Medicine 2017;13(5):675-83. doi:10.5664/jcsm.6582.

3. Rodrigues Filho JC, Neves DD, Moreira GA, Viana ADC, Jr., Araújo-Melo MH. Nocturnal oximetry in the diagnosis of obstructive sleep apnea syndrome in potentially hypoxic patients due to neuromuscular diseases. Sleep Med 2021;84:127-33 doi: 10.1016/j.sleep.2021.05.009.

4. Westenberg JN, Petrof BJ, Noel F, et al. Validation of home portable monitoring for the diagnosis of sleep-disordered breathing in adolescents and adults with neuromuscular disorders. J Clin Sleep Med 2021 doi: 10.5664/jcsm.9254.
